# Supplementary material for: An Integrated Centrifugal Degassed PDMS-Based Microfluidic Device for Serial Dilution
Source: Micromachines (Basel). 2021 Apr 23;12(5):482. doi: 10.3390/mi12050482 (PMC8145514; doi:10.3390/mi12050482)
Supplement: Supplementary file 1 [file micromachines-12-00482-s001.zip › Supplemental Materials_Final-Serial Dilution-SMALL SUNY Buffalo.docx]

**SUPPLEMENTAL MATERIAL**

An Integrated Centrifugal Degassed PDMS-Based Microfluidic Device for Serial Dilution

Anyang Wang, Samaneh Moghadasi Boroujeni, Philip J. Schneider, Liam B. Christie, Kyle A. Mancuso, Stelios T. Andreadis and Kwang W. Oh

**Figure S1.** (a) Schematic and (b) image showing the effect of the Coriolis force on the device. A rotation speed of 1400 RPM was applied counter-clockwise. Scale bar represents 3 mm. The Coriolis force density is expressed as $\vec{F_{c}}=2 \rho\vec{v}\times\vec{\omega}$. The Coriolis force is a vector cross-product of a flow velocity vector, $\vec{v}$, and an angular velocity vector, $\vec{\omega}$. Therefore, the force on the flow moving from the metering chamber to the dilution chamber with a counter-clockwise rotation will be in the opposite spin direction (e.g., clockwise). The sample interface may be not flat right after the rotation. (e.g. the blue sample in the left waste chamber) If needed, abrupt angular acceleration, deacceleration, or mechanical vibrations may be applied to flat the interface of the sample, which will lead to the smooth sample transfer to the next chamber.

**Figure S2.** Comparison between sample volume measurements by micropipettes and pixel counts. Digital micropipette aspiration was used to measure the sample volume in this paper. Manual digital micropipette-based measurements can measure with a resolution of up to 0.1 $\mu l$; however, the accuracy of the measurements is subject to manual operation skills. To ensure accuracy, 2D pixel counting using Wasabi! software (Hamamatsu Photonics, Shizuoka, Japan) was also used for comparison. The 2D pixel counting approach is reliable although it lacks height information. More than four test devices were used for measuring, respectively. In Fig. (b) and (c), volume measurement using these two approaches revealed the same trend for both measurements. In Fig. (d), the trapped liquid volume between the metering chamber and the dilution chamber was measured using 2D pixel counting. Then, the estimated volume was calculated considering the height of the channel/chamber.
